# Experiences of nurses involved in natural disaster relief: A meta‐synthesis of qualitative literature

**DOI:** 10.1111/jocn.15476

**Published:** 2020-09-17

**Authors:** Chao‐Li Xue, Yu‐Sheng Shu, Mark Hayter, Amanda Lee

**Affiliations:** ^1^ School of Nursing Yangzhou University Yangzhou City Jiangsu Province China; ^2^ Northern Jiangsu People’s Hospital Yangzhou Jiangsu China; ^3^ University of Hull Faculty of Health Sciences University of Hull Hull UK

**Keywords:** disaster management, disaster nursing, disaster relief, meta‐synthesis, natural disaster, nurses

## Abstract

**Aim:**

To explore nurses’ experiences in natural disaster response.

**Background:**

Nurses are key to disaster response. There is a growing body of qualitative research exploring this emerging nursing issue. However, there is a need to synthesise and summarise this body of knowledge to identify the overarching elements of how nurses experience working in disaster situations to reflect on their experiences so that we may help shape future clinical practice, research and education.

**Design:**

Qualitative meta‐synthesis.

**Method:**

Following PROSPERO guidelines (Moher et al., 2015), an exhaustive and systematic literature search and quality appraisal was undertaken in December 2019 to reveal nurses’ experiences during natural disaster response. Sandelowski and Barroso's systematic retrieval, analysis and interpretation of findings method was used to produce a meta‐summary of findings from 10 papers evaluating experiences across 9 disasters. A meta‐aggregation was used to synthesise the findings from the studies and was methodically quality assessed with PRISMA and CASP.

**Results:**

Our findings aggregated data from 42 sub‐themes, into the following four themes to capture nurses’ experiences after responding to disasters. These included agile response; leadership and innovative problem solving; building resilience; positive communication and need for psychological/emotional support.

**Discussion:**

This meta‐synthesis provides evidence to illustrate nurses’ resilience and leadership capabilities as means to manage and perceive their disaster relief response. Factors such as emotional intelligence, capacity to react to changing situations, to manage scant resources in extreme situations were highlighted in nurses practising in highly stressful environments. Managers can use these examples to support ways to improve disaster management policies, but also, to engage in support for their staff.

**Relevance to clinical practice:**

The role of nursing staff in disaster rescue is receiving significant attention. Understanding nurses’ experiences during disaster rescue can help future leaders to improve capacity to respond and nursing preparedness through education, training and management, but also for continuing emotional support after the event.

**What does this paper contribute to the wider global clinical community?**

- This review identifies the important role of nurses in the field of disaster rescue and proposes areas that nurses need to improve in the process of implementing disaster rescue, by synthesising the experiences of nurses who participating in natural disaster relief.
- The four themes capture cohesive directions to inform future practice, to inform disaster management strategies and to continue emotional and psychological support which helps nurses after the event.

## 1 INTRODUCTION

Professional preparation of nurses to manage disasters is critical to safe and effective rescue and response. Nurses working in disaster settings must have clear leadership, and disaster rescue policies to ensure they can react to disaster situations (Li et al., 2017). Many nurses who have reported being a part of disaster response teams experience psychological distress—exacerbated by lack of effective management, communication mechanisms and resources (Mao, Fung, Hu, & Loke, 2018). In 2019, the International Nursing Council (ICN) and the World Health Organization (WHO) jointly proposed a framework for disaster care offering statements on diagnoses, outcomes and interventions appropriate to disaster response (ICN, 2019). These capture the physiological, psychological social and environmental needs of patients and their families. However, there is limited information to evaluate how nurses cope with disasters. There are also calls for us to look more closely at how nurse experiences can be used to inform training strategies and curricula which may be specifically designed to prepare nurses to work effectively in disaster settings.

Professional nursing skills in disaster response are critical. In a time of significant global warming (Schenk, 2019), natural disasters are increasing and earthquakes, tsunami, volcanic activity, floods and landslides more commonplace. In just one decade (2007 to 2017), human deaths from natural disasters are estimated at 60,000. During the more recent coronavirus (COVID‐19) outbreak, nurses have been key to the disaster response teams, relying on their clinical skills and on heuristics so that they could adequately manage complex clinical demands in highly complex and volatile situations. All nurses have to be responsive and they must be able to provide effective disaster relief (Alpert et al., 2018). However, they need to be supported in their roles. By understanding the demands placed upon them, considering their feelings, predicting anticipated problems and appreciating expectations, we can help nurses to manage more effectively in disaster situations. To date, research has focussed on small‐scale projects to evaluate disaster experiences and responses, so it is both timely and appropriate to consider synthesising and aggregating all evidence to inform disaster management in the future.

Thus, this paper evaluates the current body of qualitative research on how nurses relate their experiences in disaster response work. It offers an in‐depth analysis of all studies which explore nurses’ experiences after having responded to a natural disaster. Meta‐synthesis as a methodology presents an objective idealistic viewpoint of the phenomenon under investigation (Sandelowski & Barosso, 2003). By capturing all qualitative evidence through systematic literature review, it classifies findings, forming a meta‐summary of themes to offer a novel interpretation of data that in turn can inform practice, generate new research agendas and inform education.

## 2 BACKGROUND

Nurses are key to disaster relief planning, response and recovery, and further exploration of their experiences is needed to inform future practice. There is a significant call for increased disaster nursing education, to improve critical thinking skills and readiness to respond (Veenema et al., 2017). Yet, education and training must be appropriate, and based on evidence drawn from reflections of nurses who have experienced disaster responses. By learning from previous disaster nurses’ experiences, educational interventions can be developed to include strategies which enhance nurses' psychological resilience. Understanding how nurses act and respond, how they communicate and identifying the most supportive leadership techniques is key to developing an awareness of how to act in the future.

There are many quantitative studies on nurses’ disaster response. However, most are evaluative studies to review educational interventions for disaster preparedness, disaster response and postdisaster management undertaken in a simulated environment. Though quantitative research reveals key elements of disaster response, it lacks the ability to offer rich detail on nurses’ experiences. Quantitative research cannot assess nurses' opinions, intentions and role awareness in disaster response (Alzahrani, 2017). It cannot adequately reflect the impact that environment, legal and organisational cultures have on nurses' ability to respond to disasters (Geum, 2017). Performance can also be shaped by morals, sociodemographic factors and peer/supervisor behaviours noted during the disaster response (Ahayalimudin and Osman, 2016). To broaden the understanding of nursing and disaster response, qualitative studies must be undertaken. However, the nature of qualitative research means smaller sample sizes, and data which may not be as transferrable to other situations. Considering the multi‐faceted phenomenon of “disaster,” many studies focus on events which are population limited and are relevant to the specific disaster, its geography and the accessibility of health services. Thus, a collation, amalgamation and meta‐synthesis of all evidence are timely.

Qualitative meta‐synthesis offers a merger of empirical evidence, developing a deeper understanding of the phenomenon, or concept under study (Downe et al., 2019). Yet, it can pose epistemological challenges, where qualitative synthesis of other researcher's data on experiences brings alternative constructs. For example, the nature of “disaster” is complex—one person's understanding of “disaster” can be—for example—a large road traffic accident, whereas another could mean full scale events such as the Tsunami disasters. Sandelowski and Barosso (2003) offer objective idealism as a philosophical standpoint, to review existing empirical research data and group them into new and innovatively aggregated themes so they optimise validity of the subject under evaluation.

Conducting a meta‐synthesis means all researchers must reach consensus in their development of constructs. For consensus, we maintained the theoretical presupposition of objective idealism. Objective idealism asserts a stance, and a “reality” which combines and transcends the natures of the object experienced (qualitative data) and of the minds of the observers. Supporting Hegel's philosophy, it offers a common sense approach to interpreting content through objective thought. With this in mind, we chose a cross cultural team of experienced researchers to undertake an exhaustive literature search, to review and form consensus opinion on the qualitative evidence. We then offer a novel interpretation—a meta‐summary—of all findings from qualitative studies on nurses’ experiences of disaster response. The methodology is derived from seminal authors Sandelowski and Barosso (2003), who use discussion, iterative processes and repetitive considerations of themes as means to extract and reanalyse data.

The study protocol was not eligible for PROSPERO registration as there are no specific health‐related outcomes anticipated from this work. However, the PROSPERO guidelines for systematic retrieval and methodology were followed.

### 2.1 Aim

To undertake a systematic review and meta‐synthesis of the qualitative evidence on nurses' experiences whilst working in natural disaster response.

### 2.2 Study design

We performed a systematic review meta‐synthesis of all published empirical qualitative data which focussed on nurses’ experience in disaster response. We applied Moher's et al. (2015) guidelines for systematic review and meta‐analysis to report results and thematic analysis through qualitative matrices. No ethical permits are required for meta‐synthesis, and no external funding was sought. The equator checklist document used in this systematic review was PRISMA, see Appendix S1.

### 2.3 Search strategy

In December 2019, authors conducted a systematic search with key MeSH and free‐text terms (Table 1). The search used seven databases: PubMed; CINAHL; Cochrane Library; Eric; PsyINFO; Scopus; and Academic Search Premier. The university librarian was consulted on Boolean operands and key search strategy.

**TABLE 1:** MeSH search terms

MeSH search terms			
#1: (nurses) or (nursing);	#2: (disaster) or (catastrophe) or (calamity);	#3: (experience) or (qualitative),	#1 & #2 & #3

Explicit inclusion and exclusion criteria were set through consensus opinion of researchers and through an iterative literature review on disaster nursing (Table 2).

**TABLE 2:** Review inclusion and exclusion criteria

Inclusion criteria	Exclusion criteria
‐ Papers written in English. ‐ Papers focusing on: Nurses’ responding experiences involving in the nature disaster relief. Nurses’ responding experiences make sense to the disaster relief. ‐ Papers with qualitative or mixed‐method studies which reporting of qualitative results.	‐ Papers not written in English. ‐ Papers focusing on other fields. ‐ Review papers, quantitative papers, discussion papers, reporting papers, position papers, grey literatures, reflection papers, editorials and commentaries. ‐ Studies with qualitative results but not focusing on the nurses’ reposing in the nature disaster events.

Sandelowski and Barosso's (2003) first two methods for integrating qualitative research findings was applied to extract all relevant qualitative findings from empirical evidence and then to reduce those findings into abstracted elements—or themes. After removal of duplicates, titles and abstracts were screened. Full‐text reviews were undertaken to identify any papers which were not relevant to the research objective, or where qualitative data could not be extracted. Finally included papers were reviewed by a team of researchers to ensure they met inclusion criteria (CZ, AL, MH). A research matrix was developed which included a list of authors, date of publication, the geography (or country), the aim, sample size and disaster type, study design, collection and analysis, limitations, quality appraisal and potential themes (Table 3). A final, independent check of the matrix was undertaken by an experienced professor of nursing research (MH).

**TABLE 3:** Description of included studies

Study	Country	Aim	Sample size; setting and type of disaster	Study design; data collection and data analysis	Limitations	CASP	Themes
Masoud and Fereshteh (2014)	Iran	To design a tool for evaluation of nurse competence for disaster response.	35 nurses with recent experiences of the medical response to disasters. Hospital. None discussed	‐ Qualitative descriptive study. ‐ Date collection: Individual interviews. Date analysis: a five‐step method of Granheim and Lundman content analysis.	The relatively small sample size limits the general applicability of the results.	Medium quality	Five main themes included: management competences; ethical and legal competences; team working; personal competences, and specific technical competences that presented in this report.
Van Devanter et al. (2017)	USA	To explore, from the nurses’ perspective, what the challenges and resources were to carrying out their responsibilities, and what the implications are for nursing education and preparation for disaster.	16 of 20 nurses contacted agreed to participate, 12 held staff nurse positions and 4 held management positions. Hospital. The storm that began in the Caribbean and moved up the east coast of the United States eventually took 149 lives and left billions of dollars in damage to communities.	‐ A mixed‐methods study included qualitative interviews with a purposive sample of nurses and an online survey of nurses. ‐ Date collection: Experienced qualitative interviewers conducted 1‐hr interviews with participants. Date analysis: a three‐step process, initially conducting open coding followed by focused coding and finally identification of major themes.	‐ Qualitative data are not generalisable to other settings. ‐ The study was conducted 6 to 10 months after Hurricane Sandy and the hospital evacuation, thus, study participants’ recall could be affected.	Medium quality	Survey data identified important resources that helped nurses to carry out their roles, including support from coworkers, providing support to others, personal resourcefulness, and leadership.
Van Devanter et al. (2014)	USA	To develop a more complete understanding of natural disasters and to contextualise the data.	16 of 20 nurses contacted agreed to participate, 12 held staff nurse positions and 4 held management positions. Hospital. A 12‐ft storm surge generated by Hurricane Sandy crossed the major highway that runs along the East River in Manhattan overwhelming three major hospitals located along First Avenue.	‐ A mixed‐methods study consisting of in‐depth qualitative interviews with a purposive sample of nurses followed by a quantitative online survey. ‐ Date collection: Experienced qualitative interviewers conducted 1‐hr interviews with participants. Date analysis: a three‐step process, initially conducting open coding followed by focused coding and finally identification of major themes.	‐ Qualitative data are not generalisable to other settings. ‐ The study was conducted 6 to 10 months after Hurricane Sandy and the hospital evacuation, thus, study participants’ recall could be affected.	Medium quality	Identified two major challenges experienced by nurses: challenges related to practice and psychosocial challenges; and two major resources that buffered the stress of the experience: peer support and supervisory support.
Zhou et al. (2015)	China	Describe the experiences of Chinese nurses who worked in disaster relief after the Wenchuan and Yushu earthquakes, and their views about future disaster nursing education/training programmes.	12 registered nurses from four hospitals in Wuhan, Hubei Province were recruited. Hospital. The Wenchuan 8.0‐magnitude earthquake caused 87,476 deaths, many injuries, and damages. The Yushu earthquake in Qinghai Province caused 2,698 deaths, and >100,000 injured.	‐ Qualitative descriptive study. ‐ Date collection: Interviews with digitally recorded in private and ranged 50−97 min. Date analysis: Riessman's narrative inquiry method.	Participants came only from hospitals in Wuhan. Nurses from other regions of China who worked in the disaster zones may have other instructive and perhaps different experiences.	High quality	Five themes emerged: unbeatable challenges; qualities of a disaster nurse; mental health and trauma; poor disaster planning and coordination; and urgently needed disaster education.
Yang et al. (2010)	China	To provide an understanding of how Chinese nurses acted in response to the 2008 Wenchuan earthquake.	10 registered nurses from three tertiary teaching hospitals affiliated with a university of Chongqing, China, they were participated in the on‐side earthquake rescue. Hospital. A devastating earthquake, measuring 8.0 on the Richter scale, struck the Wenchuan region of Sichuan province.	‐ Gadamer's philosophical hermeneutics. ‐ Date collection: A semi‐structured interview guide was used in the in‐depth interviews. Date analysis: thematic analysis.	Findings cannot be generalised because of the use of qualitative study; it only explored nurses’ experience in on‐site rescue, and the knowledge, skills and attributes demanded for disaster nurses revealed can only be partial.	High quality	Three themes were identified: feeling under‐prepared; perceived challenges and coping strategies; and the rediscovery of the helping and caring role.
Nasrabadi et al. (2007)	Iran	To explore Iranian Registered Nurses’ experiences in disaster relief in the Bam earthquake, Iran in 2003.	13 Iranian registered nurses with a bachelor's degree in nursing who had at least 2 weeks’ experience as RNs during the Bam disaster in the earthquake location. Hospital. A powerful earthquake struck south‐eastern Iran on 27 December 2003, killing more than 43,000 and injuring 20,000 people, 6,000 were left homeless, and much of the city of Bam was destroyed.	‐ Qualitative descriptive study. ‐ Date collection: a series of semi‐structured interviews conducted by the author, the interviews lasted from 45 to 90 min. Date analysis: the method of latent content analysis.	The study didn't justify their research method and described little about the data analysis.	Medium quality	Three general themes emerged: (a) the need for previously prepared practical protocols, (b) the need for qualified and real teamworking in the situation, and (c) the need to establish periodic comprehensive training programmes in disaster relief nursing.
Sloand et al. (2013)	Haiti	To explore the experience of volunteer nurses after the Haiti earthquake, January 2010.	12 nurse volunteers were purposively recruited and selected by the research team using snowball sampling. Hospital. A disastrous earthquake struck Haiti near the capital of Port‐au‐Prince. The epicentre was 15 miles from the capital with a magnitude of 7.0.	‐ Qualitative descriptive study. ‐ Date collection: semi‐structured interviews took place in person at the participants’ place of choice (*n* = 8) or by telephone (*n* = 4). Date analysis: using strategies described by Corbin and Strauss (2008).	Researcher and participant's relationship was not described; no detail information about ethics.	Medium quality	Six themes emerged: initial shock, relentless work, substituting and making do, questioning, systems building and transitioning back.
Johal and Mounsey (2015)	New Zealand	To explore how nurses are coping with the dual challenge of personal and professional impact following the earthquakes and into the recovery process.	11 nurses from across the Christchurch area, they were registered nurses who were working in Christchurch between September 2010 and February 2011. Christchurch area. A magnitude 7.1 earthquake struck the Canterbury region of New Zealand.	‐ Qualitative descriptive study. ‐ Date collection: used semi‐structured open‐ended interviews to elicit extended answers to questions about the challenges nurses have faced during and following the earthquakes. Date analysis: grounded theory approach.	None discussed.	High quality	A number of themes that are related to the concept of post‐traumatic growth including improvement in relationships with others, change in perspective or values, changed views of self and acknowledgement of the value of the experience.
Negar et al. (2017)	Iran	To explore the experience and perceptions of nurses who have served in times of disaster in Iran in order to identify their real problems and challenges.	15 participants were selected purposefully, they were all nurses who had experience in providing healthcare services at the time of nature disasters. Hospital and university. None discussed.	‐ Qualitative descriptive study. ‐ Date collection: semi‐structured interviews. Date analysis: an inductive qualitative content analysis approach; using the Lundmen and Grancheim five stages content analysis method.	None discussed.	High quality	Five main themes: afraid of probability of recurrence, necessity of providing healthcare services for an unknown period of time, challenge of what to prioritise, nurses’ own conflicting emotions, and their concern for their own families.
Nekooei et al. (2014)	Iran	To deeply and comprehensively understand the needs of nurses for aid and casualty support; To help further in understanding, constructing and applying the proper educational and technical programmes for disaster management.	23 nurses were interviewed, they were working at Kerman Medical University educational hospital, or the KMU faculty of Nursing, who had more than five years’ experience in general nursing practice. Hospital and university. None discussed.	‐ Qualitative descriptive study. ‐ Date collection: open semi‐structured individual interviews. Date analysis: content analysis.	The study didn't justify research method.	High quality	Four major themes: 1) psychological support, 2) appropriate clinical skills education, 3) appropriate disaster management, supervision and programming, and 4) the establishment of ready for action groups and emergency sites.

## 3 RESULTS

**FIGURE 1:**
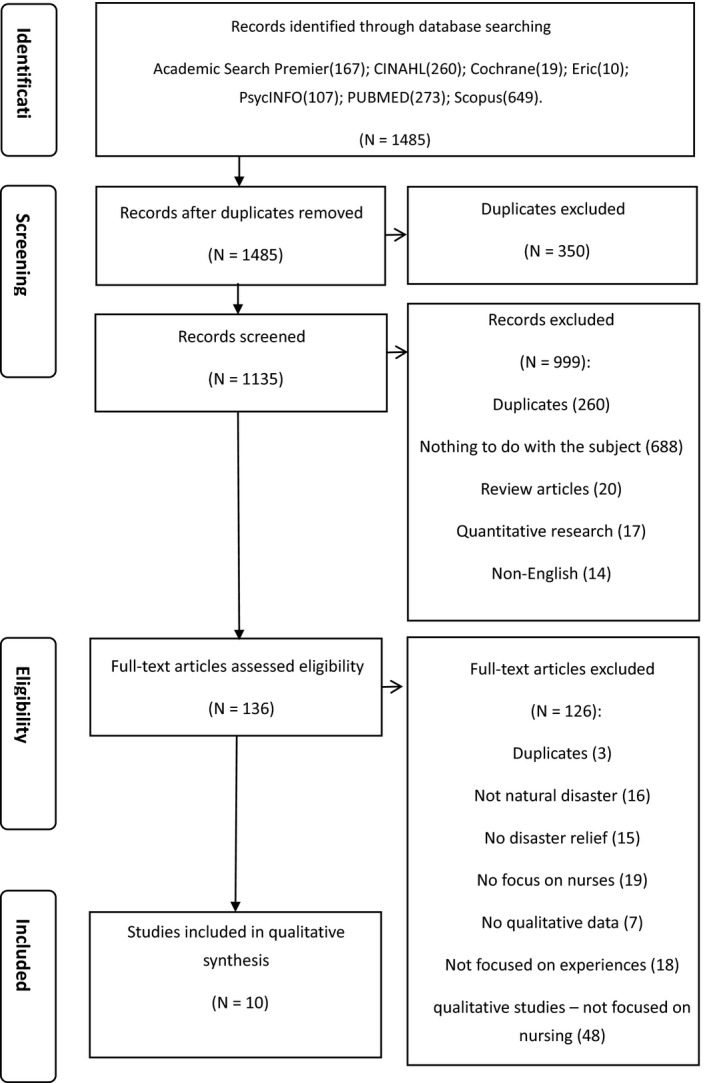
PRISMA flow diagram

Following guidelines for systematic review and meta‐analysis (Moher et al., 2015), we retrieved 1,485 papers and eliminated 350 duplicate papers through the RefWorks software. A total of 990 papers were removed on review of titles and abstract as they did not meet the research question. Full‐text reviews of 145 publications revealed 135 papers incompatible with the research topic. Finally, 10 papers which resulted from 9 disaster studies which fully met the research question were identified for inclusion in the meta‐synthesis. The PRISMA flow diagram shows results for this study (Figure 1).

### 3.1 Quality appraisal

The qualitative Critical Appraisal Skills Programme (CASP) tool was used to score research design and rigour for a comprehensive quality assessment of included papers (CASP, 2018). For this meta‐synthesis, one reviewer used the CASP tool to evaluate the included literature independently (CZ), and evaluations were compared and discussed against findings from other authors (MH, AL). Meta‐synthesis requires all studies to be included—whether graded as high or low quality, because excluding low‐quality papers can impact comprehensiveness of results. CASP, as a tool, offers a framework with no interpretivist paradigm and so was felt to be the most appropriate tool in the early phases of the study, reflecting and reasserting objective idealistic philosophical positioning stance of all authors.

### 3.2 Data abstraction and synthesis

All results were aggregated into an excel database and independently checked by all three reviewers. All reviewers collaborated to discuss, revisit and reveal themes from the findings of the studies (CZ, AL, MH). Using Sandelowski and Barroso's (2003) technique, line by line coding was applied, then themes drawn from the results. Thematic analysis was enhanced through techniques such as “word cloud and frequencies” to form descriptive and conceptual themes from categories and sub‐categories. These were drawn from narratives of the nurses who participated in all qualitative studies.

### 3.3 Study characteristics

Of the 10 included papers, 2 were studies after one disaster response in the United States of America (Van Devanter, Kovner, Raveis, McCollum, & Keller, 2014; Van Devanter, Raveis, Kovner, McCollum, & Keller, 2017), 2 from China (Zhou, Turale, Stone, & Petrini, 2015; Yang, Xiao, Cheng, Zhu, & Arbon, 2010), and 4 were from Iran (Masoud & Fereshteh, 2014; Nasrabadi, Naji, Mirzabeigi, & Dadbakhs, 2007; Nekooei, Saeed, Khanjani, & Arab, 2014; Negar, Norouzi, Ahmadi, Hosseini, & Khankeh, 2017). There was one each in Haiti (Sloand, Ho, & Kub, 2013) and New Zealand (Johal & Mounsey, 2015). The disasters included response to a storm (Van Devanter et al., 2014, 2017) and an earthquake (Zhou et al., 2015; Yang et al., 2010); (Nasrabadi et al., 2007; Sloand et al., 2013); (Johal & Mounsey, 2015). Three of the papers did not disclose the nature of disaster. Participants across all studies were nurses with disaster rescue experience who had participated in natural disaster rescue. Data collection methods across all studies included face‐to‐face and telephone interviews. A total of 7 studies were performed in hospitals, 2 across hospitals and universities, and 1 was undertaken in the Christchurch community.

### 3.4 Qualitative synthesis findings

Qualitative meta‐synthesis requires reciprocal translations for interpreting in vivo statements, so that researchers can develop new concepts which arise from original data sources. We extracted all data into an excel workbook, coding responses and theming data through discussions and consensus opinion. We aggregated four themes from a total of twenty codes to reflect narratives of nurses with experience of natural disaster relief.

Nurses after disaster relief reported initial phases of operationalising systems and logistics after the event were difficult at times, when colleagues were establishing their own professional roles and responsibilities. Narratives showed that leadership and adaptability to solve problems was essential to improved disaster management. Many nurses expressed the need for psychological support and positive communication. One main outcome through many of the narratives was that nurses reported that they had to build a resilience—to cope with the onslaught of pressures and clinical demands in (often extreme) conditions. Table 4 summarises data against the derived themes.

**TABLE 4:** Overarching themes

Theme	Summary	Data
Agile response		
(6 papers) Van Devanter et al. (2017), Van Devanter et al. (2014), Zhou et al. (2015), Nasrabadi et al. (2007), Nekooei et al. (2014), Masoud and Fereshteh (2014), CODES included Protocols, guidelines, decision‐making Prioritisation, duties, responsibilities Operationalisation Supplies, skills knowledge safety.	Many participants wanted clear protocols and guidelines to help them during their work. They reported that a lack of clear systems, processes and protocols affected their abilities to form an efficient response:	*Without protocols and guidelines, we worked hard but mostly in the dark with trial and errors. If we knew previously how we should act in a different situation, or we had some protocols on hand or in mind, we could act more effectively and more efficiently*. (Nasrabadi et al., 2007) *When we came there in the first times of the event we were confused like other people, nobody told us what we should do and from where we must begin… after a quick assessment of the affected field our group decided to start with our own decision making*. (Nasrabadi et al., 2007)
	Their response to disaster was also hindered by chaotic response by people with intentions to help—who were perhaps not a part of the rescue:	*… there were too many cars going there, and we stayed there for two days but only received one patient. (Jiu)*. (Zhou et al., 2015) *“There were many people who did not help and even prevented others from working. They were crowding and causing chaos everywhere. They wanted to help, but they were actually causing trouble for the personnel work.”* (Masoud & Fereshteh, 2014)
	Operational level responses were also a concern. Increasing numbers of hospital attendees and external factors such as power cuts – meant that triage and signposting of potential patients and their relatives required levels of logistical thought and operational management with consideration of capability and capacity to deliver required care:	*I think the most frustrating part was the communication. We didn't have phone service, our emergency phone, no electricity, no computers, we're so focused on technology now… my only way to communicate was to use my Blackberry, the telephone and our cell phones and hopefully they didn't die because we couldn't charge them… so it was communication*. (Van Devanter et al., 2017) *“Hospitals have to announce beforehand, how many patients they can accept; so that more patients are not sent there. Otherwise, patients would face problems and we would lose patients we could save.”* (Nekooei et al., 2014)
	Operationalisation, delivery and allocation of resources were also an issue for many nurses. Participants often faced a shortage of resources during disaster relief, which seriously affects the efficiency of rescue:	*There was neither sufficient equipment or medicine. Only penicillin was available, but it did not work. It was very hot inside the tent and I saw that the patient was gasping, but all I could do was fan him. (Shier)*. (Zhou et al., 2015) *Equipment and supplies for operating theatre in the tent were unavailable due to transport problems… We had to use whatever was available. We used mineral drinking water to clean instruments and iodine to disinfect the instruments instead of high‐pressure sterilization. I still question myself if the practice was right, although it saved lives in that situation*. (Yang et al., 2010)
	The need for organizational hierarchy, to map specific capabilities with staff who possess specific professional knowledge was identified as means to help problem solving	*P1: Knowledge about duties, responsibilities, and restricts is very important, and nurses should be familiar with this boundaries*. (*Masoud, 2014*) *I think organizationally that that command structure, I’m not really sure that all of it, that it really gets down to all the staff, quite honestly. I think the staff probably know what happens on their unit and they defer to the nurse manager if there's an issue on their unit,*. *.. if it's who to shut off oxygen or what to do, they go to the nurse in charge. So I think… that they probably just look at the person on their unit as opposed to the whole structure…”* (Van Devanter et al., 2017)
	Many nurses felt that knowledge on how to respond to disasters was sparse, amongst managers, nurses and communities. They wanted more information—training and advice to support a more coordinated community, health, emergency services and allied professional response to deal with natural disasters:	*In general, we had little knowledge and few skills in disaster relief nursing. I think we and the other health care providers should extend our knowledge and improve our skills in disaster relief practices*. (Nasrabadi et al., 2007) *I think nurses all across the city need to be educated about… disaster, nurses from 99 different hospitals may show up [at yours] to work… This is our expectation about how you are going to behave towards them… what you are going to do to make the transition for them easier*. (Van, 2017)
	Many participants called for further education and training	*The first thing in the education of disaster nursing is self‐protection…Another thing, (we) must establish is a sound relief process and system. There were too many people going to that place, but they did not integrate their resources well, which caused a great waste. I believe disaster education should be very good. And the only hope is that disaster training for nurses and doctors can (happen) and that this knowledge can enter schools as early as possible. (Shisan)*. (Zhou et al., 2015)
Leadership and innovative problem solving		
(7) papers Van Devanter et al. (2017), Van Devanter et al. (2014), Zhou et al., 2015 Masoud and Fereshteh (2014), Yang et al. (2010), Nasrabadi et al. (2007), Sloand et al. (2013), CODES Emotions (leaders) Sharing equipment/resources empowerment, extending roles and remits, informing and disseminating, leaders offering support	Some participants identified that they didn't always know what to do—that to cope—they needed to be flexible, adapting to role changes and complex situations during their disaster scenarios, but identified good leadership as integral to managing demands	*“I felt like I had no idea what I was doing. I don't know what my role is” (P5)*. (Van Devanter et al., 2017) *We were supported. We had staff meetings (with NYULMC supervisors) once a month while we were out that we all went to and were able to express our concerns and they were able to address the ones they could but they always heard us out…And they made sure they were there for us. P10*. (Van Devanter et al., 2014)
	Participants who relied on ‘usual working practices felt disempowered at lack of resources and infrastructure:	*that was really frustrating… [there were] a lot of cases in which we couldn't do anything and we knew that there is nothing to be done, where in the United States… we would've been able to fix those problems… but in Haiti there was just no way to fix it. (RN12)*. (Sloand et al., 2013) *There was neither sufficient equipment or medicine. Only penicillin was available, but it did not work. It was very hot inside the tent and I saw that the patient was gasping, but all I could do was fan him. (Shier)*. (Zhou et al., 2015)
	However, there were positive factors identified within narratives. Many participants applied heuristics, using their existing skills to problem solve and identify innovative solutions to manage clinical demands and prevent further problems occurring. They reported a need to act in a range of roles—(nurses, educators, resource managers, cooks and housekeepers, leaders and coordinators):	*‘‘Both work wise and personally, because I might not have had that wake‐up call. I might have thought everything just carries along and life is sweet and when you, when you're put to the test it's when you find out who you really are and what your strengths are and what you can and can't do.’’* (Johal & Mounsey, 2015) *As the local medical services were paralyzed, we took charge of medical supplies from different medical stations and negotiated with other rescue teams for sharing resources and supplies in the town… We could not gain any assistance from local healthcare professionals and we had to learn local knowledge about residents, water and food sources from our patients and their relatives… We had to work as housekeepers and search for food, cook, and arrange accommodation for the entire rescue team*. (Yang et al., 2010)
	Leadership was mentioned across various studies. Many participants mentioned the importance of establishing good relationships, communication and trust between nurses and their managers. Good leadership support allowed nurses to gain more confidence to manage during their disaster response:	*The leadership was great. Our senior leadership was there (at the command center). Our nurse manager was there… our medical director. And whatever they got from the command center, they were good at disseminating the information to us. (P4)* (Van, 2017) *First, a manager should know what causes stress in staff. Then, assess individuals’ stress level and react to them based on their stress level … Because stress is just like (a) virus; if you do not control it, everyone would be overtaken by it. I think identifying risk factors in the scene is of great importance. It is even beyond doing anything else*. (Masoud & Fereshteh, 2014)
	Nursing managers also reported the importance of communicating with their staff, reassuring and assisting staff in coping with stressful situations and increasing demands:	*I did have nurses with fears…they would text me constantly. Text messaging was a big form of communication, and that would be any hour of the night. I had one nurse breakdown one particular night and you know, just couldn't even function, couldn't even work but I was right there with her. Getting in touch with her. Making sure she was ok…P15*. (Van, 2014)
Building resilience		
Van Devanter et al. (2017), Van Devanter (2014), Zhou et al. (2015), Masoud & Fereshteh, 2014 Nekooei et al. (2014), Sloand et al. (2013) CODES Adaptation, reflecting on event, resilience, exhaustive working practices, ethics, culture of working, preparedness, experiences on site,.	Resilience is the power, or capacity to cope and rise to the challenges ahead, and a variety of participants showed how they developed that capacity, being flexible and managing the setbacks in a positive and productive way:	In the first few days of the disaster, everything is chaotic, but health care delivery is not just for one day, sometimes you have to be present at the site of the disaster for one, two, or even three months.”(Negar et al., 2017) As a nurse, that prepares you for nothing. I mean… I have all my certifications, I have all this… I’m qualified, but you're not prepared for anything like that… I mean nothing really prepares you, you just gotta adapt once you get there. (RN7). (Sloand et al., 2013)
	The qualitative narrative data illustrated how nurses had to control and improve their own skills to maintain a positive environment, but also how this helped them cope with patients and relatives’ emotional fluctuations:	… there is a disaster and although it is very dangerous, after you go there you do feel there is a shadow in your mind. But you may just think, oh dear, I went, I once went there. No matter how much my contribution was, I helped the people there. I think in my heart that feeling is still very good. Anyway, sometimes I feel myself very fragile. Really, just feel very fragile. (Yi). (Zhou et al., 2015) There's gonna be some things that you don't ever want to see but you know you're there… doing good for these people and without your emotional stability… you're just gonna have to ignore it and keep on going or you're worthless to the people there. (RN7). (Sloand et al., 2013)
	Heavy workloads were attributed to reducing resilience in disaster response. They caused conflict in work areas across the hospitals, with participants reporting being pressured by prolonged periods in practice:	We ended up working 3–4 shifts a week, 12 hr…nurses on our floor weren't happy because other floors (from NYU) didn't work at all from October to January. Just didn't work. P10.(Van, 2014) “We had 10 consecutive days working in the hospital and then I was in hospital for 10 days due to severe fatigue. A lot of nurses were sick, including me. I was hospitalized again for 10 days due to high work pressure.” (Negar et al., 2017)
	Narratives illustrated a variety of confounding factors in building resilience—such as regional and cultural differences, ethical issues, and insurmountable challenges during the rescue:	“The situation is very different in the disaster, limited resources, shortage of staff, you are not able to provide all services during a disaster, and you choose who will be the first priority to be cared for. The ethical debate is happening.” (Negar et al., 2017) But from my experience we didn't know what to expect. So, there was a bit of a culture shock associated with this, even the hospital administration may have been prepared for this but we didn't know who we have to talk to, how to get the ambulances here, they were prepared, we weren't… so in the future I think that part of nursing education… there should be a component… disaster preparedness. What happens in the event of a disaster, if you are working in a hospital, what may you be called on to do. (P2) (Van Devanter et al., 2017)
	There were many additional physiological demands. For example, a participant from China stated that on arrival to the earthquake site, she had difficulty breathing due to altitude sickness and was hypoxic, this made her nervous and “afraid.” She found it difficult to undertake the rescue mission when she felt her life was threatened:	Just going there, wow, I really felt really out of breath. Before I inhaled oxygen, I felt it was so unbearable. (Chunhua). (Zhou et al., 2015) The road was totally destroyed and we had to walk more than 9 hr to Yinxiu, carrying a 30 kg backpack. We had to give up equipment we'd prepared, including the mobile operating theatre. It is quite physically demanding… On the way to Yinxiu, we witnessed tragic life losses and brutal damages. I had a strong feeling that I was incompetent for this task… Be honest, when rocks fell during the aftershocks, I had a fear of dying in the scene. (Yang et al., 2010)
	There were also moral and ethical issues raised, which made nurses question themselves. Lack of resources and no time to consider actions and decisions left them feeling uncertain, worrying whether they were acting in the best interests of their patients:	The situation is very different in the disaster, limited resources, shortage of staff, you are not able to provide all services during a disaster, and you choose who will be the first priority to be cared for. The ethical debate is happening.” (Negar et al., 2017) They expect a minimum care from nurses and, we also tried to provide them with relatively good care, as well as we could, even though, many times this involved self denial and self sacrifice. (Nasrabadi et al., 2007)
	One participant identified that he could not implement effective on‐site evacuation, as he was unable to clearly articulate his own clinical skills with anticipated roles and tasks, which made him feel scared	I felt tired, and frustration when I was vague in the situation. I didn't know the right task that I should do while I had responsibility to carry out so many things in a stressful state. (Nasrabadi, 2007) “I couldn't imagine evacuating in the middle of a hurricane” (P10).(Van Devanter et al., 2017)
Positive communication and Psychological support		
Positive communication and Psychological support Van Devanter et al. (2017), Van Devanter et al. (2014), Yang et al. (2010), Sloand et al. (2013), Johal and Mounsey(2015), Nekooei et al. (2014) CODES After event considerations, communication strategies postevent, support for each other	Many participants felt overwhelmed, nervous and upset, highlighting the need for psychological support during disaster response	Nurses working in the aftermath of a disaster should have frequent psychological counseling, and after a shift someone has to sit down and talk to them, to make sure they are not facing (psychological) problems; are neglected and no one has anything to do with them, if nurses have psychological problems themselves, they cannot support the injured.” (Nekooei et al., 2014)
	The narratives showed particular stressors as nervousness (of new situations), fear (safety and for relatives/friends because of the disaster), having to work long hours away from family and friends meant they became reliant upon peers and contact with family members during their long working hours:	We texted, we emailed, we even created a separate Facebook group so that we had to invite each other…We tried to keep in touch as a whole group, especially in the beginning when it was so fresh and so unstable…we were scared. We were holding on to see who knew what and was everybody ok. And as things started to stabilize, things started to settle down we started to keep in touch in smaller groups…we got closer because we needed each other more…I talked to my coworkers on the phone more in those three months than I talked to them on the phone almost 5 years that I’ve worked here. P10.(Van Devanter et al., 2014)
	They became reliant upon colleagues, or local families who were also affected by the disaster	“we counselled each other, I think, at work” (Johal & Mounsey, 2015) “we had friends in other parts of Christchurch where they would just invite us round for tea…we just had the support of family and friends and it was okay.” (Johal & Mounsey, 2015)
	There was a heavy reliance on telecommunications, stating use of electronic information technology was crucial so that they could continue communicating with their disaster response teams but also—their families:	Due to the interruption of telecommunications we lost contact with our families for quite a long period, and we even did not know if we could survive when we were threatened by aftershocks. We totally depended on the team support in such an uncertain period. We felt we became close and cared for each other. (Yang et al., 2010) “All of us were worried about our houses; because of the shortage of nurses we had to stay in the hospital, but we were constantly worried, the telephones were disconnected so we could not receive any news from our family.” (Negar et al., 2017)
	A variety of participants reported how important their friends were for psychological (and physiological) support during the disaster responses:	We received on‐site psychological counseling services from professional counselors who joined our rescue team a few days later and worked side‐by‐side with us… They distributed information booklets that established how to cope with trauma situations. They also organized group discussions to help us identify and report early signs and symptoms of posttraumatic stress disorder. (Yang et al., 2010) Every single person that worked in any kind of a department here… like the guys in the suits were up here with hard hats and jeans on and everybody in the world seemed like was up here helping us to move.(P7) (Van Devanter et al., 2017)

The four themes offer an overview of nurses experiences in disaster management. Most papers offered data to support these themes, but they did offer a different focus. Some papers in our study focussed on competence, coordination and coping strategies (emotional and physical), whereas others looked to evaluate “perceived challenges” or aimed to support education/supervision and provision of competencies. The range of methods to evaluate nurses’ experiences in disaster relief appears to depend on a number of factors, including the nature of disaster and its geographical setting. Some papers presented their themes in terms of how to action future events, whereas others framed themes in terms of emotional and psychological effects.

This paper offers overarching themes to capture all information across studies. A total six papers contained data which offered information on agility in response—offering themes which aligned with factors such as “legislation, competencies, coordination of site, and preparedness.” There were seven papers which identified leadership and innovations in problem solving. These papers included themes such as building and capturing systems, innovating and being flexible/adaptable. They noted the importance of empowerment and/or decision‐making—and related this to having good leadership, communication and trust across networks. Data linked to building resilience were found in six of the papers. This theme captures how nurses adapted and managed their emotions, their actions and found capacity to cope with the onslaught of events. The final theme assimilates data on how nurses perceived communication, how they felt they needed support and relied upon colleagues and peers. This theme captures the essence of nurses’ feelings across the total duration of disaster response. Although all studies identified personal communication and needs for support, only 6 papers offered data to support nurses’ views.

## 4 DISCUSSION

This study offers a meta‐synthesis of qualitative data following a systematic search. Although all evidence should be considered for meta‐synthesis (even poor‐quality research publications) the methodological quality of all included papers was rated as “moderate or higher” through CASP (2017).

This qualitative meta‐synthesis study extracted four main themes by analysing the experience of nurses in participating in natural disaster relief. Other current and existing literature reported on disaster management and relief.

### 4.1 Agile response – This includes—operationalisation postevent—resource management, professional response and healthcare provision across networks

There are country level professional expectations and frameworks which allow nurses to practice (e.g. the Nursing and Midwifery Council (NMC) in the UK, General Nursing Councils and regulatory of America, National Health and Family Planning Commission—China). When any nurse reports for response to a disaster, all the necessary checks must be in place to ensure critical and appropriate response for all participants.

The implementation of disaster rescue by nurses depends on the guidance of regional disaster emergency plans and agreements. It involves the entire process of disaster rescue and directly affects the rescue order and the resilience of the recovery work. International Disaster Law (Aronsson‐Storrier and da Costa, 2017) clearly states that national legislation is an important aspect of supporting disaster management, and legislation has a guiding role in any disaster prevention work or rescue operation.

Participants identified “chaos” at disaster scenes severely affected their ability to perform rescues. In addition, they sited lack of available resources and equipment failure caused by the disaster as important factors which hindered rescue operations (N. Sattler, Claramita, & Muskavage, 2018). There were power outages, causing electrical equipment to fail. Participants reported this increased workload and reduced clinical efficiency.

Thus, it is important to evaluate the hospital's capacity to deal with the emergency. Based on the actual situation in the local area, hospital managers should cooperate with government departments and organise multi‐agency cooperation and operation (Mills, Helm, Jola ‐Sanchez, Tatikonda, & Courtney, 2018). These multi‐agency networks should formulate scientific and feasible disaster relief protocols and guidelines, and promulgate laws and regulations on disaster management; thus, they can support hospitals' ability to respond to disasters and oversee comprehensive disaster response.

Operationalisation and management of resources was highlighted as significant to the nurses’ ability to respond. This is supported by Rodríguez, Albores, and Brewster (2018), who has identified the need to allocate resources based on perceived needs analysis of the particular disaster site; thus, waste is reduced and resources are appropriate to the situation. Logistical interoperability of all the different organisations is essential to ensure resources are adequate, can get to the site of need and do not impinge on the disaster response.

Cranmer identifies the specialist logistical expertise required (Cranmer and Aschkenasay, 2014). He asserts that disaster response requires a comprehensive set of knowledge, skills and logistics management and suggests that a complete disaster deployment framework must be established—but this should also include provisions for both physical and mental health care. This is important, because all out qualitative data revealed nurses needed emotional support to maintain resilience. Cranmer raised the need for previous professional experience, team‐based response, flexible disaster relief skills and good communication skills which reflected the cultural background of nurses who were conducting relief tasks.

Interdisciplinary, information technology managed and “cross‐networked” health care is another important aspect of supporting disaster management. Incorporating information and electronic medical technology into disaster management can improve communication and workflow between managers and healthcare providers (Norris et al., 2015). This was important to the nurses in our study—as many participants identified use of IT for implementing rescue, but also seeking support and guidance from peers and their families.

Some of the participants across these qualitative studies identified the need for streamlining decision‐making through protocols and guidelines. Disaster protocols offer a theoretical basis for disaster managers so that they can ensure clarity of procedures and clinical activities (Mills et al., 2018). When deploying rescue missions, they can provide novice nurses with some degree of orientation to the rescue.

The construction of the disaster agreement is based on the collaboration of multiple people from different disciplines and institutions; they have accumulated a lot of rescue experience during the implementation of the rescue in the disaster area and can propose a chronological organisation of effective disasters, including formulating predisaster plans, disaster relief processes and postdisaster evacuation strategy (Wilson et al., 2015).

Thus, protocols and guidelines may be useful for nurses both during and after disaster relief. The disaster protocol can be applied to evaluate feasibility of early warning systems, help managers evaluate the model, content and structure of disaster management and refine the key elements in the field, so as to provide guidance for the establishment of a new comprehensive early warning system (Khankeh, Hosseini, Farrokhi, Hosseini, & Amanat, 2019). Research shows that disaster emergency response operating systems are the influencing factors for nurses to implement disaster relief, including disaster relief protocols and guidelines, legal systems for disaster management and hospital's emergency capacity building. Participants said that the disaster relief protocols and guidelines can provide orientation guidance for nurses to implement rescue, which is consistent with the research that imperfect emergency response operating systems often caused disaster scenes to become more chaotic.

Many disasters lead to damage to telecommunication systems, and the optimisation of network protocols in disaster management can improve communication efficiency in disaster‐stricken integrated heterogeneous networks (Kawamoto, Nishiyama, & Kato, 2012). Although disaster protocols have educated nurses on their rescue efforts, Masoud noted that results are not always positive. Some nurses, despite training in disaster relief, still feel overwhelmed when facing real disaster scenarios. Thus, there may be an active dissonance between (protocol driven theory of how it “should be done”) versus the actual situation during disaster response.

### 4.2 Leadership and innovative problem solving

Nurse leaders’ decision‐making directly impacts on the quality of nursing and the safety of patients. The sharp rise of the ability of nursing managers to lead the crisis and organise the response to mass casualty incidents highlights the important role of leadership in disaster relief (Veenema, 2017). Disaster rescue is a systemic task that requires teamwork to find effective solutions to meet needs.

Nursing leadership officially guides nurses to work in solidarity and cooperation (Smith, Nelson, & Porter, 2019). It is very important for the implementation of disaster management to protect the interests of nurses and respect their own values during the rescue. Transformational leadership has a positive impact on employees' behaviours. It promotes employees' self‐efficacy, enhances problem‐solving and promotes innovative behaviour development—because transformational nurse leaders act by stimulating their teams’ internal motivation.

Good nursing leadership provides nurses with moderate freedom of work, encourages a sense of collegiality within the nursing team and actively supports nurses’ clinical work. It is cyclical, because it enhances the collective sense, improves managers’ trust and encourages more confidence to work effectively (Amankwaa, Gyensare, & Susomrith, 2019).

This is reflected in our findings. Nurse leadership was cited as highly important to effective disaster relief across all the qualitative data. Participants stated that the shortage of rescue supplies under disaster conditions severely limited rescue efficiency. Nursing leaders, as front‐line guides, are obliged to make accurate judgments in a timely manner based on the rescue situation, formulate resource emergency plans, reasonably allocate supplies to different rescue areas and ensure that nurses have enough supplies.

Participants reported feeling uncomfortable with lack of appropriate resources, unreasonable workload distribution and fuzzy accountability networks. This is supported by Pazirandeh and Maghsoudi (2018) paper which identified the need for nurse leaders to consider effective coordination of staff and adequate resource management. They must also establish mutual and trustworthy relationships with all teams so that they support clinically effective caregiving during disasters.

Further studies identify trust as key to disaster responses (Afsar and Masood, 2018). Good leaders can avoid uncertainty, engage trust, break down silo mentality and encourage innovative work behaviours, whilst being sympathetic to their staff. Participants across all these qualitative studies identified the need for good leadership and trust amongst colleagues. A study by Kerfoot (2019) went further, identifying a need for nurse leaders to develop emergency plans, cultivate specific nursing teams so that they can adequately respond to disasters. Kerfoot also acknowledges the need for nursing leaders to continuously improve their professional skills so they can guide nurses to implement effective rescue in crisis.

Research shows that nurses' ability to adapt and solve problems is a key aspect of disaster relief. Some participants claimed that nurses could not complete even the most basic rescue tasks due to their inability to adapt to changing roles, this is consistent with the findings of Thomas's and Niels (2016) research. Thomas identified that lack of rescue experience and lack of disaster knowledge and skills meant nurses were ill prepared.

This situation is alarming, because nurses are a significant force to maintain and contain public health risks. Thus, it is nurses who should be integral to forming national health policies and disaster risk management strategies (Kanbara, Yamamoto, Sugishita, Nakasa, & Moriguchi, 2017). In our meta‐synthesis, many participants reported they were able to adapt quickly to changing roles, expanding their role and scope of practice to suit disaster scenarios. They acted as leaders, educators, responders and decision makers.

Thus, nurses should strengthen national dialogue with public service organisations to improve responses to disaster relief (Veenema, 2016). However, many participants in our meta‐synthesis expressed a need for further education. Xia, Li, Chen, Jin, and Zhang (2019) identifies some basic skills, such as triage methods and basic life support technologies to assist in disaster response. Most nurses stated that they had not received disaster training and believed that disaster knowledge education and online training, especially the disaster drills advocated by interactive forums, were very important for nurses to implement disaster relief. This is supported by Sonneborn, Miller, Head, and Cross (2018).

Disaster drills may be optimal to improving quality of rescue for nurses. Simulation enables nurses to clearly define roles and responsibilities during rescue, and it strengthens professional theoretical knowledge. However, this is refuted by Nekooei et al. (2014), who identified that disaster training drills do not always bring positive results, because of the multi‐faceted nature of disaster.

It may be reasonable to assume that hospital managers should set training courses to assist disaster preparedness of nurses, as all nurses have a duty to continuously strengthen their professional knowledge and engage in learning.

### 4.3 Building resilience

“Resilient Nurses” can cope with challenges in disaster relief, and this includes fulfilling basic needs, maintaining an emotional stability, being self‐confident, engaging in social support, seeking and reflecting on truth, insights and beliefs. There were many examples of how nurses developed their resilience across all the studies, how they provided innovative solutions to meet their patients and relatives’ basic needs, but also engages strategies to meet their own. Participants mentioned their resilience was affected by many factors, such as regional cultural differences, ethical issues and unmet challenges in the disaster relief. Nurses often felt constrained by humanitarian ethics when they made decisions during the disaster rescue and this is supported by quantitative studies which included in this study. These authors raise issues such as resource allocation, patient classification, treatment priorities, cultural differences and concepts of “fair treatment”—which impacted decisive decision‐making and impaired resilience to adapt to changing situations.

Our data assimilation identified several coping strategies, where nurses “slept in extreme conditions” and endured difficult physical environments. They adopted strategies to maintain levels of physical health so that they could manage and sustain their caregiving. It is very important to maintain physical and mental health so the quality of rescue is sustainable (Kester and Wei, 2018). Helping nurses build resilience enables them to accurately perceive stress and make effective coping strategies, especially during disaster relief, nurses face multiple uncertainties, transitional and restructuring conditions, it is necessary for them to develop resilience.

There are many reasons that should be considered when building resilience, such as establishing self‐esteem, maintaining supportive social relationships, maintaining flexibility, focusing on one's own needs, solving problems in a timely manner, setting reasonable goals and maintaining a positive attitude (Davies, 2019). Cultivating resilience can enhance the personal characteristics of nurses, enable nurses to respond to various emergencies more effectively in the rescue and improve work flexibility and adaptability; it also promotes nurses to be good at discovering their strengths during the work and formulating rescue strategies based on their own circumstances, strengthening communication between teams and helping to build cohesive professional resilience. In addition, the resilience also has the potential to remind, guide, enlighten and heal, it can effectively help nurses to maintain positive work beliefs in adversity, reduce occupational dislocations caused by overload stress and help maintain normal functions and continue work (Cope, Jones, & Hendricks, 2016).

To build resilience amongst the nursing workforce, leaders must effectively coordinate rescue tasks, arrange nurses' work schedules reasonably, organise nurses to receive education on disaster events and the health risks caused by disaster and provide nurses with a flexible resilience buffer period in disaster relief. Therefore, to improve nurses' resilience in disaster relief, hospital managers must provide targeted education to guide nurses on ethical decisions (Greco, Lewis, Sanford, Sawin, & Ames, 2019). Laws and policies must support nurses’ response in disaster relief to reduce physical, psychological and emotional risks to these primary caregivers.

### 4.4 Positive communication and psychological support

Many participant responses across all qualitative studies showed an increase in psychological pressure. Lee, Lee, Park, and Lee (2017) has identified that these pressures arise from difficulties in cooperation with new team members, bad disaster experiences and the potential for severe consequences of disaster. Brooks (Brooks, Dunn, Amlôt, Greenberg, & Rubin, 2016) identified a number of predisaster factors which can be associated with negative disaster response. These include occupation, individual disaster preparedness and training experience, life events and health status. Factors during the disaster include disaster exposure, duration of time spent at the disaster site, emotional state and surrounding trauma effects, role‐related sources of stress, perception of hazards and risks and external support. The postdisaster factors include professional support, impact on life and coping strategies.

These stressors are important, because they impact on nurses’ ability to implement disaster relief. The complexity of disaster events and the urgency of rescue work mean nurses have significant psychological pressures during rescue processes. Many nurses experience vicarious traumatisation and form subsequent emotional troubles after disaster response (Corcoran, 2020). Therefore, psychological counselling for nurses is important, throughout the entire disaster relief operation. Disaster management agencies and leaders should actively guide nurses to implement self‐care supporting postdisaster psychological rehabilitation and improving self‐efficacy. Managers must also implement effective measures to improve nurses' job satisfaction and social support through social activities and sharing of rescue experiences. Nurses should also encourage relatives and friends to provide companion support for optimal mental health and multi‐system communication.

Participants claimed that nurses were easily to develop negative emotional and psychological stress with the overworked and persistent stressful of mind in disaster scenarios, and they will face different levels of psychological and emotional problems during and after the disaster. Without timely intervention, this can develop into post‐traumatic stress syndrome, which not only affects the nurses’ physical and mental health, but also severely hinders the progress of disaster rescue.

Some participants also indicated that they would continue to worry about recurrence for a long time after the disaster. Many questioned their future careers because of the fear of rescue work. This may be because of a lack of communication and psychological support.

Online communication based on electronic information technology can provide a sustainable communication support for nurses who cannot communicate face‐to‐face due to the pressure of working hours. The use of electronic information equipment can promote communication between team members and help nurses relieve stress by sharing rescue experiences. Nurses’ psychological health is related to daily life, working environment and stress, risk perception and acquiring knowledge of coping with risk control methods (Nukui et al., 2017). Nursing managers should actively maintain effective communication with disaster relief nurses and implement one‐to‐one communication support for psychologically vulnerable nurses. They must also cultivate nurses to maintain mental health awareness.

### 4.5 Limitations

This study has limitations. Although the researchers searched seven databases systematically, they did not rule out that related studies from other sources were omitted; also, this study only included English literature, and studies on other languages have not been included, which may cause some of the main research results to be missing. However, the study adopts a strict article review process to ensure the quality control of the research and ensure that the results are scientific.

## 5 CONCLUSION

This qualitative meta‐synthesis highlights some crucial factors to inform leadership and education in disaster nursing and we can use this to shape future practice. There are both short‐term and longer term impacts of disaster management on nurses and continued psychological support is crucial.

Nurses feel they can offer more agile response to disasters, when empowered by good leadership and support. Positive communication is key to building resilience and helps nurses to find innovative solutions during real‐time events.

Future research should focus on the development of interventions to improve nurses' emergency response capabilities, broaden the scope of qualitative research on disaster relief for nurses, and extract more relevant themes to promote the development of nurses in disaster relief, fully refer to the opinions of nurses to build the disaster management system and perfect the important role of nursing in disaster rescue.

## 6 RELEVANCE TO CLINICAL PRACTICE

This review amalgamates the qualitative literature on nurse's experiences of working in disaster areas. There are several key messages for practice. First, the importance of communication and planning and the value of keeping in touch with practitioners through whatever means to ensure plans and instructions are disseminated. Second, the need to equip nurses with problem‐solving skills and the ability to think flexibly and imaginatively when plans and communication are reduced and when situations are fluid. These skills can be learnt and should be part of training for nurses in disaster relief work. Nurse managers need to ensure that those working in disaster areas have access to psychological support—both the long and short term. Longer term may involve face‐to‐face counselling, whereas shorter term could be online access to emotional support structures. Third, the review illustrates the importance of resilience. Building confidence and self‐belief needs to part of the problem‐solving and disaster relief training for nurses. As a global cadre of nurses with these experiences increase, we should use them more extensively in the training and development of those practitioners yet to face such a challenging work environment.

## CONFLICT OF INTEREST

None.

## Supporting information

Appendix S1
